# Type 2 diabetes impacts colorectal adenoma detection in screening colonoscopy

**DOI:** 10.1038/s41598-020-64344-2

**Published:** 2020-05-08

**Authors:** Lorenzo F. Ottaviano, Xueying Li, Matthew Murray, Jesse T. Frye, Brandon E. Lung, Ying Yi Zhang, Jie Yang, Erin M. Taub, Juan Carlos Bucobo, Jonathan M. Buscaglia, Ellen Li, Joshua D. Miller

**Affiliations:** 1 Department of Medicine, Renaissance School of Medicine at Stony Brook University, Stony Brook, New York, United States of America; 2 Department of Family, Population and Preventive Health, Renaissance School of Medicine at Stony Brook University, Stony Brook, New York, United States of America

**Keywords:** Diabetes, Colorectal cancer

## Abstract

Background: Diabetes is associated with an increased risk of colorectal cancer (CRC). We conducted a retrospective analysis of adenoma detection rates (ADR) in initial screening colonoscopies to further investigate the role of diabetes in adenoma detection. Methods: A chart review was performed on initial average risk screening colonoscopies (ages 45–75) during 2012–2015. Data collected included basic demographics, insurance, BMI, family history of CRC, smoking, diabetes, and aspirin use. Multivariable generalized linear mixed models for binary outcomes were used to examine the relationship between diabetes and variables associated with CRC risk and ADR. Results: Of 2865 screening colonoscopies, 282 were performed on patients with type 2 diabetes (T2DM). Multivariable analysis suggested that T2DM (OR = 1.49, 95% CI:1.13–1.97, p = 0.0047) was associated with an increased ADR, as well as smoking, older age, higher BMI and male sex (all p < 0.05). For patients with T2DM, those not taking diabetes medications were more likely to have an adenoma than those taking medication (OR = 2.38, 95% CI:1.09–5.2, p = 0.03). Conclusion: T2DM has an effect on ADR after controlling for multiple confounding variables. Early interventions for prevention of T2DM and prescribing anti-diabetes medications may reduce development of colonic adenomas and may contribute to CRC prevention.

## Introduction

Colorectal Cancer (CRC) is the fourth most common cancer in the United States (US) and second most lethal^1^. Most colon cancers develop via a multistep process involving a series of somatic genetic mutations and histopathologic changes that accumulate over time that is estimated to take approximately 10–15 years^2^. Optical colonoscopy screening has been considered the gold standard for CRC screening, because of its ability to view the entire colon and to both detect and remove polyps during the same procedure, which have been demonstrated to reduce the subsequent risk of CRC^3^. However, despite the effectiveness of CRC screening it is estimated 50,680 people in the US will die in 2018 from CRC^4^.

Identifying and understanding risk factors and conditions that increase the risk of CRC is essential to optimizing the effectiveness of CRC screening. There are many established risk factors for CRC: age, race, male sex, family history in a first degree relative, smoking, and BMI^5,6^. Studies have also shown that individuals with diabetes mellitus have an increased risk of developing CRC when compared to patients without the disease^7^. This is important as diabetes mellitus is estimated to affect 9.4% of the population, or approximately 30.3 million people in the US, with type 2 diabetes accounting for 90 to 95% of diabetes cases^8^. To address the question as to how diabetes mellitus increases the risk of CRC, studies have been conducted to address whether the association with the disease also exists for precursor stages along the adenoma-carcinoma progression pathway, by examining the association between diabetes mellitus and adenoma detection rate (ADR) (detection of ≥1 adenoma). While the results have been mixed^9–13^, particularly one study conducted in Black/African American women^9^, a recent meta-analysis indicated that diabetes mellitus is associated with an increased ADR and increased advanced adenoma detection rate (AADR)^10^. One of the drawbacks of the meta-analysis is that potential confounding variables were included to varying degrees in the studies analyzed. A more recent study examining a random selection of 1800 colonoscopies in a single cohort, did not detect a significant effect of diabetes mellitus when controlled for confounding variables^12^.

To further examine the role of type 2 diabetes in colorectal adenoma-carcinoma progression, we conducted a retrospective analysis of all initial screening colonoscopies performed at a single institution, while attempting to control for confounding variables, including those previously associated with increased risk of CRC. In addition, we analyzed the effect of anti-diabetes medication and glycemic control as measured by fasting plasma glucose (FPG) (measured on the day of the procedure) and hemoglobin A1c (HbA1c) (measured within twelve months of the procedure). We report the results of this analysis, which confirms that type 2 diabetes is associated with increased ADR while controlling for confounding variables, including BMI.

## Methods

### Collection of clinical data from initial screening colonoscopies performed in 2012–2015

This retrospective cohort study was approved by the Stony Brook University Institutional Review Board (IRB #180023) and all methods were performed in accordance with the guidelines and regulations of Stony Brook University IRB. A wavier of consent was obtained by Stony Brook University IRB for retrospective collection and analysis for deidentified demographic and medical data. Patients who underwent screening colonoscopies at Stony Brook University Hospital from January 1, 2012 to December 31, 2015, were identified using endoscopy reporting software. Patients age <45 y or >75 y, a history of previous colonoscopy, a history of inflammatory bowel diseases, known hereditary colorectal syndromes, detection of microscopic or macroscopic blood in stool and other alarm syndromes, detection of colonic masses or polyps on previous studies, were excluded from this analysis. Colonoscopies that were incomplete (did not reach the cecum) and those associated with poor bowel preparation were also excluded. Clinical metadata was manually collected on each patient using the electronic medical record (EMR) as documented at the time of screening colonoscopy and included: (1) age at time of initial screening colonoscopy (year); (2) sex (Male, Female); (3) race (White/European Ancestry, Black/African-Ancestry, Asian, Other); (4) Ethnicity (Non-Hispanic, Hispanic); (5) Insurance (Commercial, Medicare, Medicaid, Self-pay); (6) BMI (kg/m^2^); (7) Tobacco Exposure (Current within one year, Past greater than one year, Never); (8) Family history of CRC in a 1^st^ degree family member (Yes, No); (9) Documented diagnosis of diabetes (Yes, No).

For patients with diabetes mellitus, further data was collected using the EMR as documented at the time of screening colonoscopy and included: (1) FPG on day of screening colonoscopy; (2) HbA1c within 12 months of the procedure; (3) Insulin use (Yes, No); (4) Metformin use (Yes, No); (5) Sulfonylurea use (Yes, No) (6) Thiazolidinedione use (Yes, No); (7) Meglitinide use (Yes, No); (8) GLP-1 Agonist use (Yes, No); (9) DPP-4 inhibitor use (Yes, No); and (10) SGLT-2 inhibitor use (Yes, No); (11) Acarbose use (Yes, No).

The colonoscopy report was reviewed to determine if a colonic biopsy or polypectomy was performed and number of polyps detected. If a biopsy or polypectomy was performed the pathology report was reviewed to collect data on polyp type (hyperplastic vs. adenomatous). If an adenomatous polyp was removed further descriptive data was collected to characterize size and location (right colon vs. left colon). We defined a right colonic adenoma as arising from cecum, ascending colon, hepatic flexure, or transverse colon and left colonic adenoma as arising from splenic flexure, descending colon, sigmoid colon, rectosigmoid, or rectum. For each adenomatous polyp we collected data on polyp histology (tubular, tubulovillous, villous), presence of dysplasia (low grade, high grade), and adeno-carcinoma (yes or no). We defined an advanced adenoma as any adenomatous polyp containing at least one of the following features: villous or tubulovillous histology, high grade dysplasia, or size ≥1 cm. We defined a high risk adenoma as any adenomatous polyp containing at least one of the following features: villous or tubulovillous histology, high grade dysplasia, size ≥1 cm, or presence of ≥3 adenomatous polyps.

### Statistical analysis

Statistical analysis was performed utilizing the Biostatistics and Bioinformatics Share Resource at the Stony Brook University Cancer Center. Demographics were compared between diabetes and non-diabetes patients using either Wilcoxon rank sum test for continuous variables, and chi-square test with exact p-values from Monte-Carlo simulations for categorical variables. Univariate generalized linear mixed models (GLMM) were used to examine the marginal association between two clinical outcomes, adenoma detection and advanced adenoma detection, and patients’ characteristics (diabetes diagnosis, smoking, family history of first degree relative, age, gender, race, ethnicity, BMI, insurance, trainee involvement) for the entire patient population (except 8 patients with type 1 diabetes), between two clinical outcomes and patients’ characteristics (smoking, family history of first degree relative, age, gender, race, ethnicity, BMI, insurance, fellow involvement, HbA1c, glucose, and anti-diabetic medication use) for patients with type 2 diabetes. Since patients that were treated by the same physician were natural clusters, physician was considered as random effect. Multivariable GLMM with physician as random effect was used to examine the relationship between potential risk factors and adenoma detection and advance adenoma detection for the entire patient population (except 8 patients with type 1 diabetes). For adenoma detection and advanced adenoma detection in subgroup analysis for patients with type 2 diabetes, only the factors that were significant based on univariate GLMMs were further considered in the multivariable GLMMs with physician as random effect. In each multivariable regression analysis, an OR > 1 indicates that one category has more risk of having adenoma detection than the reference category, and OR < 1 indicates that one category has less risk of having adenoma detection than the reference category^14^. A logistic regression model was used to examine the relationship between FPG and glycemic control based on HbA1c level among patients with type 2 diabetes who had both glucose and HbA1c results. ROC analysis was used to examine how well pre-procedure FPG predicts glycemic control. Statistical analysis was performed using SAS 9.4 and significance level was set at 0.05 (SAS Institute Inc., Cary, NC). All authors had access to the study data and have reviewed and approved the final manuscript.

## Results

A total of 5395 screening colonoscopy reports (January 1, 2012- December 31, 2015) were identified using the endoscopy reporting software at Stony Brook University Hospital (SBUH), a New York state funded academic medical center in Long Island, New York. Manual curation of the endoscopy reports was conducted with review of the linked EMRs (clinical notes, laboratory values and pathology reports) and led to the exclusion of patients that had undergone a previous screening colonoscopy, patients that reported rectal bleeding, patients with a positive fecal occult blood test, patients with a known hereditary CRC syndrome, or patients age <45 y and ≥76 y. From a total of 2984 initial screening colonoscopies performed on patients age ≥45 y or ≤75 years, an additional 119 initial screening colonoscopies were excluded from this analysis because they were incomplete exams and/or had a poor bowel preparation.

As shown in Table 1, 282 (9.8%) of the patients had type 2 diabetes and 7 (2.4%) had type 1 diabetes. Patients were predominantly White/European Ancestry (EA). Differences in patient characteristics between the patients with and without diabetes included median age, sex, race, BMI, smoking status, aspirin use, quality of colonoscopic prep and insurance status, but not Hispanic ethnicity or family history of CRC in a first degree relative.

**Table 1:** Comparison of patient characteristics between diabetes and non-diabetes patients.

	Diabetesn = 289	Non-diabetesn = 2576	P-value
Age (y) median ± IQR	57.4 ± 10.0	54.1 ± 8.7	<0.0001
Male sex (%)	159 (55.0%)	1151 (44.7%)	0.0001
Race (%)			<0.0001
White/European Ancestry (EA)	223 (77.2%)	2271 (88.2%)	
Black/African Ancestry (AA)	29 (10.0%)	140 (5.4%)	
Asian	16 (5.5%)	72 (2.8%)	
Other	21 (7.3%)	93 (3.6%)	
Hispanic ethnicity (%)	24 (8.3%)	197 (7.6%)	0.74
Family history of CRC	11 (3.8%)	142 (5.5%)	0.27
Median BMI (kg/m^2^) ± IQR	31.2 ± 9.1	27.3 ± 6.6	<0.0001
Smoking (%)			0.0015
Current (within a year)	43 (14.9%)	333 (12.9%)	
Quit (at least a year)	105 (36.3%)	710 (27.6%)	
Never	141 (48.8%)	1533 (59.5%)	
Aspirin use (%)^3^	87 (30.1%)	273 (10.6%)	<0.0001
Fellow involvement (%)	50 (17.3%)	476 (18.5%)	0.63
Quality of colonoscopic prep			0.0002
Excellent	47 (16.3%)	604 (23.4%)	
Good	198 (68.5%)	1730 (67.2%)	
Fair	26 (9.0%)	108 (4.2%)	
Undocumented	18 (6.2%)	134 (5.2%)	
Insurance (%)			<0.0001
Commercial	167 (57.8%)	1857 (72.1%)	
Medicare	42 (14.5%)	244 (9.5%)	
Medicaid	71 (24.6%)	423 (16.4%)	
Self-pay	9 (3.1%)	52 (2.0%)	
Diabetes Type			
Type I	7 (2.4%)		
Type 2	282 (97.6%)		
Median FPG (mg/dl) ± IQR^1^	129 ± 51		
Median HbA1c % ± IQR^2^	7.2 ± 1.5 (55 mmol/mol)		
Anti-diabetes medications (%)			
None	30 (10.4%)		
Insulin	59 (20.4%)		
Metformin	190 (65.7%)		
Sulfonylurea	73 (25.3%)		
DPP4 inhibitors	38 (13.1%)		
Thiazolidinedione	10 (3.5%)		
GLP1 agonists	5 (1.7%)		
SGLT-2 inhibitor	3 (1.0%)		
Meglitinides	1 (0.3%)		
Acarbose	0 (0%)		

^1^FPS missing values = 16 (5.5%). ^2^HbA1c missing values = 143 (49.5%). ^3^Asprin use missing values = 9.

Significant P-values are **bolded**.

Patients with diabetes had a median FPG level of 129 ± 51 mg/dl. HbA1c levels were documented within the SBUH EMR in only approximately half of the patients, possibly because many of the patients were followed by primary physicians or endocrinologists outside of the institution where results were not available. Of the recorded HbA1c levels, the median HbA1c was 7.2 ± 1.5% (55 mmol/mol). The number of patients with diabetes with HbA1c > 9% (75 mmol/mol) deemed a threshold value for poor glycemic control, was 17 (11.64% of 146 patients with recorded HbA1c)^15^. The number of patients with HbA1c < 8% (64 mmol/mol) moderate control, was 110 (75.34% of the 146 patients with recorded HbA1c). The number of patients with HbA1c < 7% (53 mmol/mol) good control, was 65 (44.52% of the 146 patients with recorded HbA1c). Although there was a significant correlation between pre-procedure FPG and HbA1c (p = 0.0001) in the 141 subjects with both values measured/available, HbA1c classification of poor glycemic control could not be well predicted by glucose level alone (AUC = 0.73 with 95% CI: 0.64–0.81).

The treatment of patients with diabetes is summarized in Table 1; 10.4% of patients were on no diabetes medications. Patients were often treated with more than one anti-diabetes medication. For example, of the 59 (20.4%) patients with diabetes treated with insulin, 29 (10.03%) were also on additional oral diabetes agents. Of the 190 (65.7%) patients with diabetes treated with metformin, 82 (28.37%) were on other oral agents and/or insulin. Of the 73 (25.3%) patients with diabetes on a sulfonylurea, 58 (20.07%) were on other oral agents (most commonly metformin) and/or insulin. Of the 38 (13.1%) patients with diabetes treated with DPP4 inhibitors, 32 (11.07%) were on other oral agents and/or insulin.

The colonoscopic outcomes with respect to the type and location of polyps are summarized in Table 2. Patients with diabetes had a significantly higher prevalence of adenomas and advanced adenomas than those without diabetes. However, the distribution of the polyps with respect to colon location or number of adenomas, did not differ significantly between the two groups.

**Table 2:** Comparison of types and locations of polyps in diabetes and non-diabetes patients.

**Variables**		**Diabetes** n = 289	**Non-diabetes** n = 2576	**P-value**
Adenoma	≥1 polyp	121 (41.9%)	692 (26.9%)	**<0.0001**
Location of adenoma	Left-sided only Right-sided only Both	53 (43.8%)^a^ 46 (38.0%) 22 (18.2%)	271 (39.2%) 297 (42.9%) 124 (17.9%)	0.56
Non-serrated adenoma vs. Sessile serrated/Traditional serrated adenomas	Non-serrated adenomas Sessile serrated/Traditional serrated adenomas	116 (95.9%)^a^ 5/0 (4.1%)	655 (94.7%) 36/1 (5.3%)	0.66
Advanced adenoma		40 (33.1.0%)^a^	205 (29.6%)	0.45
High risk adenoma		51 (42.1%)^a^	250 (36.1%)	0.22
Carcinoma		1 (0.8%)^a^	2 (0.3%)	0.37
Hyperplastic polyp only		31 (10.7%)	269 (10.4%)	0.92

^a^Percentages of patients with only adenomas.

Multivariable analysis, which took into consideration potential confounding factors associated with an increased risk of colonic neoplasia (see Table 3) suggests that type 2 diabetes was significantly associated with a higher ADR (OR = 1.49, 95% CI: 1.13–1.97, p = 0.0047). In addition, multivariable analysis identified older age, male sex, smoking and increased BMI was associated with a higher ADR. Type 2 diabetes was not significantly associated with AADR after taking into consideration multiple confounding variables (OR = 1.28, 95% CI: 0.85–1.93, p = 0.24, see Table 4). However older age, male sex, smoking and increased BMI were also significantly associated with an increased AADR. For patients with type 2 diabetes, patients who were not taking diabetes medications were more likely to have an adenoma than patients who were taking diabetes medication (OR = 2.38, 95% CI: 1.09–5.2, p = 0.03). No other variables were significantly associated with ADR or AADR among patients with type 2 diabetes.

**Table 3:** Multivariable analysis of adenoma detection rate (ADR) on initial screening colonoscopy performed on patients with type 2 diabetes and non-diabetes patients.

**Variable**	**Levels**	**Odds ratio**	**95% CI**	**P-value**
Type 2 diabetes	Type 2 diabetes vs. non-diabetes	1.49	1.13–1.97	**0.0047**
BMI kg/m²	Every 1 unit increase	1.02	1–1.03	**0.027**
Smoking	Current vs. Never Current vs. Quit	1.44 1.22	1.12–1.86 0.92–1.61	**0.012**
Age (y)	Every 1 year increase	1.05	1.03–1.06	**<0.0001**
Sex	Male vs Female	1.96	1.64–2.34	**<0.0001**
Race	Black/AA vs. White/EA	0.79	0.55–1.16	0.57
	Asian vs. White/EA	1.12	0.68–1.85	
	Other vs. White/EA	0.86	0.52–1.43	
Ethnicity	Hispanic vs. non-Hispanic	0.9	0.62–1.32	0.59
Family History of CRC	History vs. no history	1.02	0.69–1.5	0.94
Aspirin use	Yes vs No	0.94	0.73–1.21	0.63
Quality of colonoscopic prep	Good vs. Excellent	1.31	1.05–1.64	0.054
	Fair vs. Excellent	1	0.64–1.55	
	Undocumented vs. Excellent	1.01	0.65–1.56	
Fellow Involvement	Yes vs. No	1.08	0.86–1.36	0.5
Insurance	Commercial vs. Medicare	1.21	0.89–1.65	0.095
	Commercial vs. Medicaid	0.84	0.66–1.07	
	Commercial vs. Self-Pay	0.6	0.33–1.08	

The estimated odds ratio and their 95% confidence intervals (CI) for the effect of each covariation on ADR were based on a multivariable generalized linear mixed model (GLMM) considering attending physician as a random effect. The p-values were based on type 3 analysis from the multivariable GLMM model. The p-values <0.05 are bolded.

**Table 4:** Multivariable analysis of advanced adenoma detection rate (AADR) on initial screening colonoscopy performed on type 2 diabetes and non-diabetes patients.

Variable	Levels	Odds ratio	95% CI	P-value
Type 2 diabetes	Type 2 diabetes vs. non-diabetes	1.28	0.85–1.93	0.24
BMI kg/m²	Every 1 unit increase	1.03	1.01–1.05	**0.0096**
Smoking	Current vs. Never	1.94	1.34–2.81	**0.0019**
	Current vs. Quit	1.62	1.09–2.42	
Age (y)	Every 1 year increase	1.05	1.03–1.08	**<0.0001**
Sex	Male vs Female	2.4	1.79–3.22	**<0.0001**
Race	Black/AA vs. White/EA	0.83	0.47–1.5	0.55
	Asian vs. White/EA	0.45	0.14–1.47	
	Other vs. White/EA	0.89	0.36–2.18	
Hispanic	Hispanic vs. non-Hispanic	0.6	0.3–1.2	0.15
Family History of CRC	History vs. no history	1.16	0.64–2.13	0.62
Aspirin use	Yes vs No	0.74	0.49–1.11	0.15
Quality of colonoscopic prep	Good vs. Excellent	1.32	0.92–1.9	0.33
	Fair vs. Excellent	0.98	0.49–1.97	
	Undocumented vs. Excellent	1.56	0.82–2.94	
Fellow Involvement	Yes vs. No	1.11	0.78–1.58	0.58
Insurance	Commercial vs. Medicare	1.13	0.71–1.8	0.24
	Commercial vs. Medicaid	0.72	0.5–1.04	
	Commercial vs. Self-Pay	0.63	0.25–1.59	

The estimated odds ratio and their 95% confidence intervals (CI) for each covariate were based on a fixed multivariable generalized linear mixed (GLMM) model on ADR. The p-values were based on type 3 analysis from the multivariable GLMM model. The p-values <0.05 are bolded.

## Discussion

In designing this retrospective study, a great deal of effort was made to accurately record and thus control for multiple confounding covariables that have been previously associated with increased CRC risk, to address the effect of diabetes on colorectal adenoma-carcinoma progression. Because operators may designate colonoscopies as screening rather than diagnostic, despite presence of a positive fecal occult blood test or history of rectal bleeding, the linked EMRs were carefully reviewed to exclude these cases. This is because the ADR in patients with a positive fecal occult blood test is significantly higher and inclusion of these patients would likely skew the outcome we were using to measure colorectal adenoma-carcinoma progression^16,17^. Similarly, care was made to exclude surveillance colonoscopies by carefully reviewing all the pathology reports in the EMR to eliminate any surveillance colonoscopies labeled as screening colonoscopies, which could also potentially skew the ADR outcome^18^. Only initial screening colonoscopies were included, because repeat screening colonoscopies were performed with varying intervals ranging from 5–10 years which could also skew the probability of finding a neoplastic lesion. We opted to study the entire cohort rather than selecting only certain reports using a case control design in order to avoid possible selection bias.

Since ADR is used as a quality metric, a major confounding variable was operator dependence. A review of Medicare data revealed that colonoscopies performed on Black/AA patients were more likely to be performed by operators with low polyp detection. Individual operator ADR have been closely monitored in the SBUH endoscopy suite, which participates in the American Society of Gastrointestinal Endoscopy (ASGE) Endoscopy Unit Recognition Program (EURP). The analyses were conducted with physicians clustered as a random variable to control for operator dependence.

The results suggest that type 2 diabetes is significantly associated with an increased risk of detecting at least one adenoma. We were not able to determine the effect of glycemic control in this study because of missing HbA1c values and because only a minority of patients were documented to have poor control. Onitillo et al^19^. reported that they detected no association between glycemic control and colorectal cancer risk and suggested that hyperinsulinemia rather than hyperglycemia was contributing to increased colorectal cancer risk. In this study, there was no significant effect of metformin use on ADR (p = 0.12) although we detected a higher ADR among patients with type 2 diabetes not taking anti-diabetes medications compared to those who were treated. In addition, we detected two potentially modifiable variables, increased BMI and smoking, as significantly associated with both increased ADR and AADR. These results support developing prospective observational studies to measure fasting insulin levels and HbA1c in all patients undergoing initial screening colonoscopy to determine whether insulin resistance and/or hyperinsulinemia contribute to increased risk of developing adenomas. Future research studies are needed to better understand colorectal carcinogenesis. One promising direction is molecular pathologic epidemiology (MPE) which can provide unique opportunities to examine the influence of diet, lifestyle, and environmental exposures on specific pathways of adenoma carcinoma progression^20^.
